# Ocular Complications in Patients on Highly Active Antiretroviral Therapy: A Case Report

**DOI:** 10.7759/cureus.47242

**Published:** 2023-10-18

**Authors:** Shovna Dash, Soumya Kanta Mohanty, Gayatree Mohanty

**Affiliations:** 1 Ophthalmology, Kalinga Institute of Medical Sciences, Bhubaneswar, IND

**Keywords:** stevens-johnson syndrome (sjs), immune recovery uveitis, dry eye, highly active antiretroviral treatment, acquired immune deficiency syndrome

## Abstract

Our article aims to report the ocular adverse effects of highly active antiretroviral therapy (HAART). In case 1, a 26-year-old male patient presented with a diminution of vision. In the absence of active cytomegalovirus (CMV) retinitis and a surge in CD4 count of more than 100 cells/µL over four months, he was diagnosed as a case of immune recovery uveitis (IRU). He responded well to topical steroids and cycloplegics. In case 2, while dry eye is a common adverse effect of HAART, our 53-year-old female patient progressed to a visually distressing stage of keratoconjunctivitis sicca. She responded to lubricants and continues to be on the same. In case 3, a 14-year-old female patient’s vision succumbed to Stevens-Johnson syndrome due to nevirapine in the absence of timely intervention. Though uncommon, debilitating ocular adverse effects may be seen with HAART. Further studies and reporting are required for an increased awareness among physicians and patients.

## Introduction

An enhancement of immune function and a resultant decrease in systemic and ocular complications of HIV is the desired action of HAART. This improved immune function may result in intraocular inflammation, called “immune recovery uveitis” (IRU), leading to vision-limiting complications. Also, most of the antiretroviral therapies cause a tear film dysfunction, which can hamper a patient’s vision. Nevirapine is widely prescribed because of its efficacy and tolerability. In rare cases, nevirapine can lead to Stevens-Johnson syndrome (SJS). This study aims to report ocular complications due to highly active antiretroviral therapy (HAART). This is a series of three cases who presented with ocular complaints following the institution of HAART. For our observational study, after approval by the institutional ethics committee and well-informed consent, patients were subjected to detailed history taking and ocular examination.

## Case presentation

Case 1

**Figure 1:**
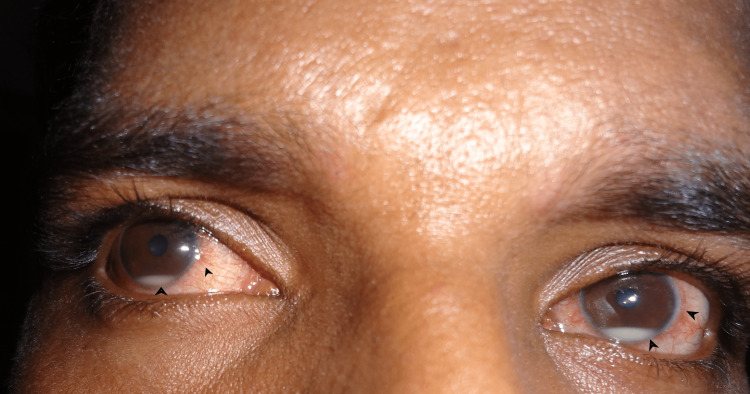
Circumcorneal congestion and bilateral hypopyon

A 26-year-old male presented to our outpatient department with a diminution of vision in both eyes for 15 days, associated with redness, ocular pain, photophobia, and floaters. He was diagnosed with human immunodeficiency virus acquired immune deficiency syndrome (HIV-AIDS) and has been on HAART for the past four months. The CD4 count was 167 cells/µL at the time of diagnosis. The patient gave no history of ocular complaints in the past. At the time of presentation, both eyes had circumcorneal congestion, mobile hypopyon (OD: 3 mm and OS: 4 mm) (Figure 1), grade 4+ aqueous cells, and fine keratic precipitates. His pupils were sluggishly reacting to light. A fundoscopy revealed mild vitritis of grade 1+, presence of vitreous strands, and mild cystoid macular edema (CME) in both eyes. An old healed cytomegalovirus (CMV) retinitis scar was seen in the inferonasal quadrant of the left eye, with no change in the size of the scar over the past four months. The best corrected visual acuity (BCVA) was 20/60 in both eyes. His systemic examination was unremarkable. The CD4 count at the time of evaluation in our clinic was 348 cells/µL.

In the absence of active CMV retinitis and a surge in the CD4 count of more than 100 cells/µL over four months, he was diagnosed as a case of IRU. The patient was started on topical prednisolone acetate 1% every two hours for two weeks, topical cycloplegics thrice a day, and topical NSAID in twice-daily dosing. With the gradual tapering of topical steroids over six weeks, the clearing of hypopyon and subsidence of uveitis were noted.

Case 2

**Figure 2:**
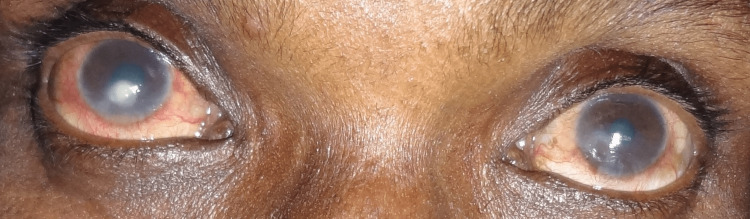
Generalized xerosis of skin with bilateral crusting of lid margins, mild conjunctival congestion, and dry lusterless cornea

**Figure 3:**
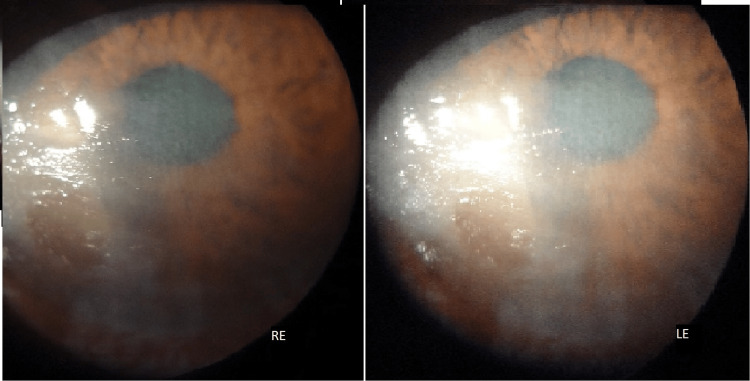
Slit-lamp examination of both RE and LE revealing multiple erosions with grayish-white mucous plaques and macular corneal opacities RE, right eye; LE, left eye

A 53-year-old female presented to us with grittiness, a burning sensation in both eyes, blurring of vision, and redness for one year. The patient was diagnosed with HIV-AIDS and has been on HAART for the past three years. She was emaciated with generalized xerosis of the skin. A complete ocular examination of both eyes revealed lid margin crusting, mild keratinization, and congestion of the conjunctiva. The corneal surface was dry, irregular, and lusterless with macular corneal opacities (Figure 2) and multiple fluorescein-positive erosions. Grayish-white mucous plaques were present over the cornea (Figure 3).

Schirmer’s test 1 revealed 3 mm wetting at 5 min. The patient had a bilateral immature cataract. The BCVA was OD: 20/80 and OS: 20/120. The CD4 count was 291 cells/µL. Taking into note, in the absence of any ocular complaints prior to the institution of HAART, the patient was diagnosed with HAART-induced severe dry-eye syndrome and was prescribed artificial tear substitutes in both drop and gel form and soft contact lenses. On the six-month follow-up, Schirmer’s test 1 readings were 7 mm (OD) and 6 mm (OS) with a vision of OD: 20/40 and OS: 20/80.

Case 3

**Figure 4:**
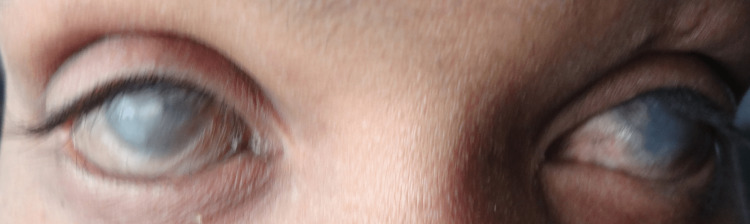
Severe dry eyes with aberrant and misdirected eyelashes

**Figure 5:**
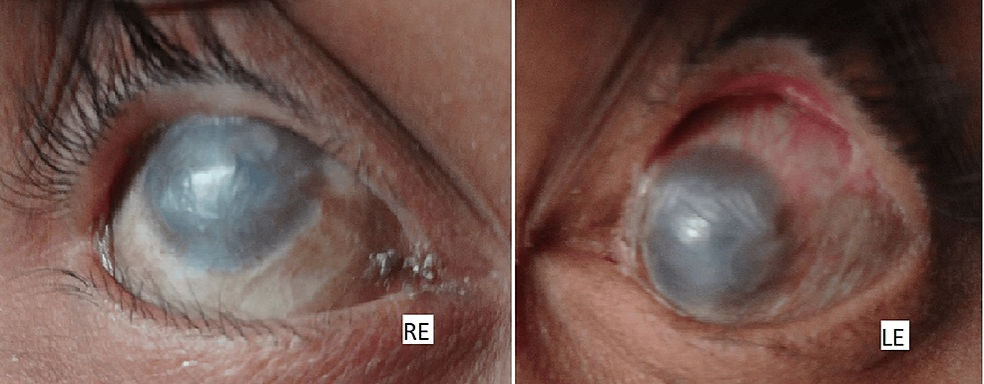
Slit-lamp examination of both RE and LE revealing conjunctival and corneal xerosis, symblepharon bands, and keratopathy, including scarring, vascularization, and keratinization RE, right eye; LE, left eye

A 14-year-old female presented with a diminution of vision in both eyes for five months. The patient was detected with HIV-AIDS six months back and started on HAART. Past records revealed it to be a nevirapine-based HAART regimen of three drugs, the other two being stavudine and lamivudine. Twenty days after the initiation of treatment, the patient presented to a peripheral health center with diffuse, exfoliating exanthema with generalized eruptions over the body. Considering the findings of mucosal involvement and skin involvement of less than 10% of the body surface area, the patient was diagnosed with nevirapine-induced SJS by the physician, and the HAART regimen was immediately withheld. The patient improved with the subsidence of symptoms. No ophthalmology consultation could be done during this acute episode due to residence in the extreme periphery and non-affordability for conveyance to a higher center. After recovery from the episode of adverse drug reaction, the patient was started again on the same HAART regimen, wherein nevirapine was substituted with efavirenz. No recurrence of similar incidents has been noted since then. At the time of presentation to our center, the patient had severe dry eyes (Figure 4). Symblepharon bands were noted in both eyes, along with keratopathy, including scarring, vascularization, and keratinization (Figures 5a, 5b).

Lashes were seen to be aberrant and misdirected. The BCVA in both eyes is as follows: perception of light is positive and projection of rays is positive in all quadrants. Hepatic enzymes were elevated, and the CD4 count was 194 cells/µL. For the keratoconjunctivitis sicca, the patient was prescribed tear substitutes, with further advice on corneal rehabilitative procedures.

## Discussion

HAART has transformed the scenario of HIV-AIDS by bringing about a dramatic decrease in morbidity and mortality, as well as a substantial improvement in ocular manifestations of AIDS. However, to the eyes, it is a bane as well as a boon, considering the ocular side effects of antiretroviral drugs.

With the advent of HAART, the longevity of HIV-AIDS patients has improved due to a successful restoration of immune function and a resultant decrease in opportunistic infections. However, this enhancement in the immune system results in a simultaneous increase in intraocular inflammation, presenting with uveitis known as “immune recovery uveitis” [1]. The incidence is estimated to be 5-30% of patients with pre-existing CMV retinitis starting HAART [2]. Such a non-infective inflammatory reaction often develops in HIV-AIDS patients having inactive CMV retinitis, following a surge in CD4 levels after initiation of HAART [3]. The current definition of IRU includes at least five main criteria: (a) being a patient with AIDS, (b) receiving HAART, (c) achieving an immune reconstitution indicated by increased CD4+ T-cell count over 100 cells/mm3 for at least two months, (d) having pre-existing CMV retinitis that is currently in the inactive state, and (e) developing an intraocular inflammation that cannot be explained by drug toxicity or a new opportunistic infection [1]. CME has been observed to be a leading cause of deterioration in vision as a part of IRU [4]. Though this condition is amenable to treatment with corticosteroids, routine supervision of vision in patients on HAART is essential.

Similarly, dry eye is noted in the majority of patients on HAART. It is usually a part of generalized dryness of the body, and for the same, not much literature is available. Though a massive decline in the incidence of opportunistic infections due to HIV has been noted after the institution of HAART, a reduction in the severity of keratoconjunctivitis sicca has not been noted [5]. This has a great impact on the patient’s quality of life [6]. It’s not just the discomfort but the visual disturbance consequent to dry eye that often leads to non-compliance to medication. In a developing country, compliance to medication rests a lot on affordability, tolerability, and efficacy of the medication.

Nevirapine, being both cheap and efficient, is widely prescribed [7]. Adverse reactions to nevirapine may range from a mild skin rash to severe hepatotoxicity [8]. However, the toxicity may go to the extremes of the spectrum like SJS. SJS or toxic epidermal necrolysis (TEN) has been reported to occur in 0.3% of patients taking nevirapine within the first four to six weeks of treatment [9]. Hence, keen vigilance and emergency management of any untoward side effects are necessary.

## Conclusions

HIV-AIDS is a highly debilitating disease that involves multiple organs. HAART has brought about a drastic reduction in these involvements, thereby decreasing morbidity and mortality alike. But with all the improvements consequent to the institution of HAART come certain adverse reactions to these medications. We aim to stress on a routine ophthalmological evaluation of patients on HAART. This shall monitor post-HAART complications like conjunctival and retinal microvasculopathy, zoster ophthalmicus, and molluscum contagiosum. Keen vigilance and emergency management of any untoward side effects like SJS are also necessary, as timely management can prevent a loss of vision. In addition, the field of pharmacogenomics needs to be explored to define patients who are susceptible to such adverse reactions. A multidisciplinary involvement in the management of patients on HAART, with routine ophthalmological evaluation, is the need of the hour.
